# Elemental and Mineralogical Composition of the Western Andean Snow (18°S–41°S)

**DOI:** 10.1038/s41598-019-44516-5

**Published:** 2019-05-31

**Authors:** Juan A. Alfonso, Raul R. Cordero, Penny M. Rowe, Steven Neshyba, Gino Casassa, Jorge Carrasco, Shelley MacDonell, Fabrice Lambert, Jaime Pizarro, Francisco Fernandoy, Sarah Feron, Alessandro Damiani, Pedro Llanillo, Edgardo Sepulveda, Jose Jorquera, Belkis Garcia, Juan M. Carrera, Pedro Oyola, Choong-Min Kang

**Affiliations:** 1 Universidad de Santiago, Av. B. O’Higgins 3363, Santiago, Chile; 2 Instituto Venezolano de Investigaciones Científicas (IVIC), Apartado 20632, Caracas, 20632 Venezuela; 3 NorthWest Research Associates, Redmond, USA; 4 Department of Chemistry, University of Puget Sound, Tacoma, USA; 5 Unidad de Glaciología y Nieves, Ministerio de Obras Públicas, Santiago, Chile; 6 Centro GAIA Antártica, Universidad de Magallanes, Punta Arenas, Chile; 7 Centro de Estudios Avanzados en Zonas Áridas (CEAZA), La Serena, Chile; 8 Department of Physical Geography, Pontificia Universidad Católica de Chile, Santiago, Chile; 9 Center for Climate and Resilience Research, Universidad de Chile, Santiago, Chile; 10 Universidad Nacional Andrés Bello, Viña del Mar, Chile; 11 School of Earth, Energy and Environmental Sciences, Stanford University, Stanford, USA; 12 Center for Environmental Remote Sensing, Chiba University, Chiba, Japan; 13 Centro Mario Molina, Antonio Bellet 292, Santiago, Chile; 14 Harvard School of Public Health (HSPH), Boston, Massachusetts, USA

**Keywords:** Hydrology, Environmental impact

## Abstract

The snowpack is an important source of water for many Andean communities. Because of its importance, elemental and mineralogical composition analysis of the Andean snow is a worthwhile effort. In this study, we conducted a chemical composition analysis (major and trace elements, mineralogy, and chemical enrichment) of surface snow sampled at 21 sites across a transect of about 2,500 km in the Chilean Andes (18–41°S). Our results enabled us to identify five depositional environments: (i) sites 1–3 (in the Atacama Desert, 18–26°S) with relatively high concentrations of metals, high abundance of quartz and low presence of arsenates, (ii) sites 4–8 (in northern Chile, 29–32°S) with relatively high abundance of quartz and low presence of metals and arsenates, (iii) sites 9–12 (in central Chile, 33–35°S) with anthropogenic enrichment of metals, relatively high values of quartz and low abundance of arsenates, (iv) sites 13–14 (also in central Chile, 35–37°S) with relatively high values of quartz and low presence of metals and arsenates, and v) sites 15–21 (in southern Chile, 37–41°S) with relatively high abundance of arsenates and low presence of metals and quartz. We found significant anthropogenic enrichment at sites close to Santiago (a major city of 6 million inhabitants) and in the Atacama Desert (that hosts several major copper mines).

## Introduction

Elemental and mineralogical composition of snow allows detecting deposition of pollutants and particulate matter^1^. Several studies have conducted chemical composition analysis of snow samples in Europe^2–5^, North America^6,7^ and Asia^8–11^. However, efforts that have targeted the Andes^12–14^ have been mostly conducted in snow on glacier surfaces at few points. Further analysis are needed, especially at snow-covered areas close to Santiago, Chile, a city of 6 million inhabitants (~33°S), with high levels of urban pollution and aerosols^15,16^.

Multiple climate regions can be identified along the Andes^17–19^. In our study area, the climate is modulated by the permanent influence of the South Pacific Anticyclonic (SPA) circulation that causes drier conditions in the Atacama Desert (18–32°S)^20^. The prevalent westerly circulation and the seasonal displacement of the SPA cause drier conditions in summer and wet conditions in winter in northern Chile (29–32°S), as well as in central Chile (33–37°S). Mountain snow precipitation associated with passing frontal systems (and cut-off lows) occurs mainly during the wet season (mid-April to September)^20^. Westerly circulation prevails in southern Chile (37–41°S) where frontal weather systems travel eastward generating snow precipitation as they cross the Andes^21^. The snowpack is an important source of water for many Andean communities in central Chile (33–37°S), where also agriculture and power generation depend on the Andean streams^22^.

The snowpack in central and southern Chile (33–41°S) has been affected by mid-tropospheric warming^23^ as well as by a persistent drought in 2010–2015^24^ that has resulted in decreasing river flows^25^. In addition to anomalies in precipitation, the Andean snowpack (18–41°S) has been impacted by particulate matter transported by near-surface winds flowing from the valleys west of the Andes towards the mountains^26,27^. The prevailing circulation affecting the high Andean mountains is mainly westerly airflow, ranging from northwesterly to southwesterly direction^21^. The subsidence inversion (strengthened by the SPA and the local topography) confines the particles within the boundary layer in the Atacama Desert and central Chile. However, the same factors lead to near-surface local circulations (i.e. mountain-valley breeze), which enable particle transport/dispersion from sources (such as major mines) that operate over the Andes^27^.

Emissions from transport, industrial pollution and residential heating in Santiago (~33°S) have been found to affect the Andean snowpack^14,26^. However, the extent of anthropogenic enrichment in the Andean snowpack remains to be thoroughly tested. In the case of the Atacama Desert and central Chile, potential sources affecting the Andean snow include the mining industry^13^; Chile is the top producer of copper and molybdenum; it accounts for approximately one third of the global copper production while ranks third in molybdenum production^28^. In the case of southern Chile, impurities in the snow may result from wood stoves (the preferred heating system for the population in that area)^29^ as well as from volcanic ash deposition^30^. Indeed, dozen of volcanoes are active in the Chilean Andes^31,32^. Because of the importance of the Andean snow for Chile, elemental and mineralogical composition analysis of the snowpack is a worthwhile effort.

**Figure 1:**
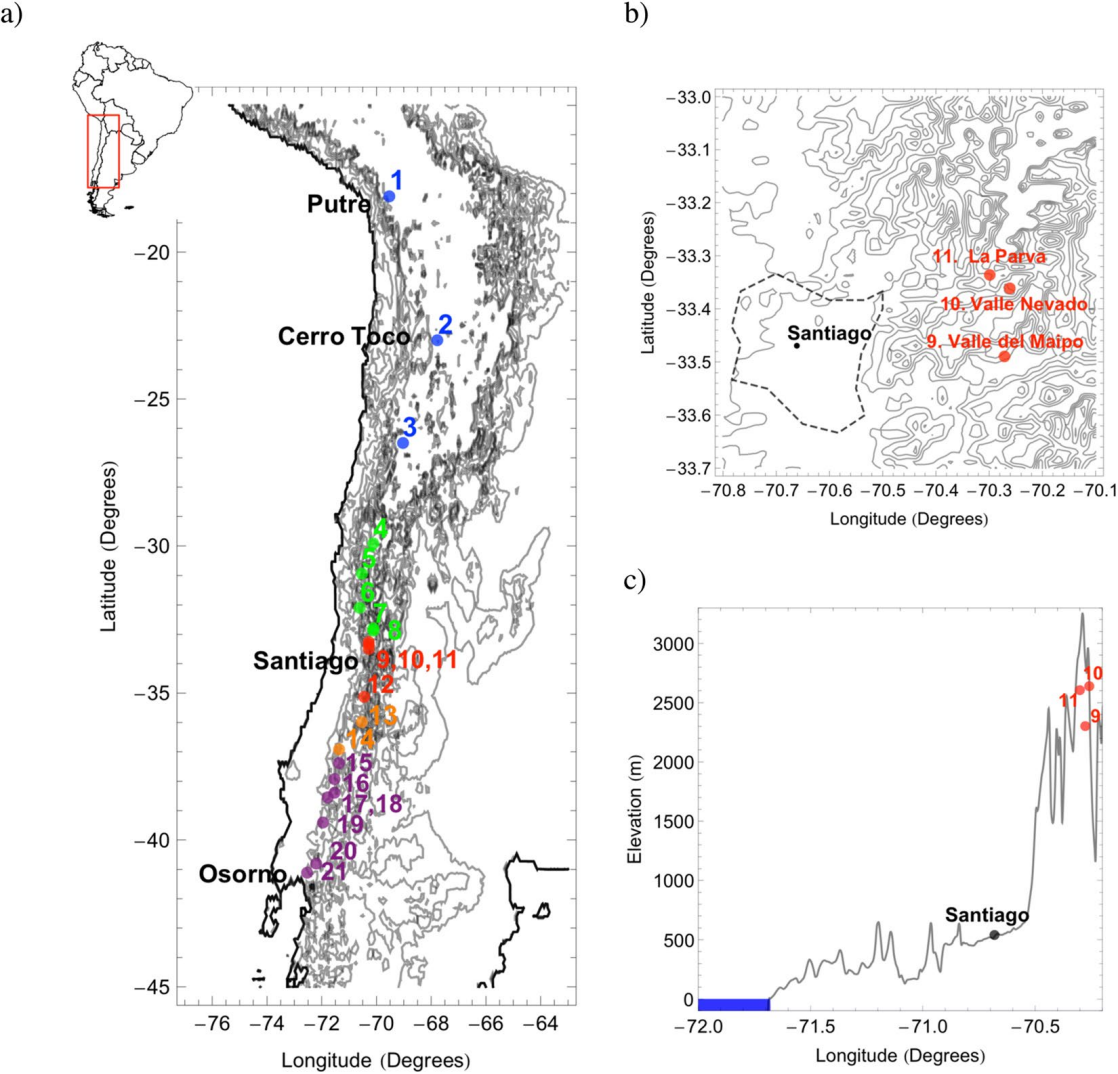
(**a**) Sampling Sites. Numbers identify the 21 sampling sites indicated in Table 1. Color code indicates the five different depositional environments identified in this study. (**b**) Sampling sites 9–11 nearby Santiago (33°27′S; –70°41′W); dotted line indicates the metropolitan area of this major city. (**c**) Elevation along latitude 33°27′S. Plots generated by using PYTHON’s Matplotlib Library (https://matplotlib.org)^62^.

In this paper, we report on the chemical composition analysis (major and trace elements, mineralogy, and chemical enrichment) of surface snow sampled during consecutive Austral winters (2015 and 2016) across a transect of about 2,500 km in the Chilean Andes (18–41°S). By combining the elemental and mineralogical composition of the snow samples, we were able to identify the five depositional environments shown in Fig. 1: (i) sites 1–3 (in the Atacama Desert) with relatively high presence of metals and quartz and low abundance of arsenates, (ii) sites 4–8 (in northern Chile,) with relatively high abundance of quartz and low presence of metals and arsenates, (iii) sites 9–12 (in central Chile) with anthropogenic enrichment of metals, relatively high values of quartz and low abundance of arsenates, (iv) sites 13–14 (also in central Chile) with relatively high values of quartz and low presence of metals and arsenates, and (v) sites 15–21 (in southern Chile) with relatively high abundance of arsenates and low presence of metals and quartz. As explained below, modest anthropogenic influence found in northern Chile and southern Chile suggests that element concentrations measured at sites in these zones may be useful as baselines for future studies.

## Material and Methods

### Sampling

Sampling was conducted during consecutive Austral winters (2015 and 2016) at 21 sites across a transect in the Chilean Andes, from Putre to Volcán Osorno; see Table 1 and Fig. 1 for further details. Sampling was carried out at the end of the accumulation season. Sampling sites were selected to represent broad regions at locations with no known local aerosol sources. At each site, two samples from the surface layer (up to 15–20 cm depth) and separated by 1–2 m distance were collected. Samples ranged from 1 to 2 kg each.

**Table 1:** Sampling sites.

Sites	Location	Latitude	Longitude	Elevation (m)	Sampling date
1	Putre	S 18°06′08.5″	W 69° 31′14.3″	5318	2015/07/05
2	Cerro Toco	S 22°57′09.4″	W 67°46′38.1″	5370	2015/07/10
3	La Ola	S 26°27′17.1″	W 69°03′0.72″	3578	2015/07/14
4	Valle del Elqui	S 29°20′42.6″	W 70°04′17.9″	2341	2015/07/17
5	La Ramada	S 30°57′38.1″	W 70°33′21.2″	1757	2015/07/19
6	Valle Choapa	S 32°03′49.2″	W 70°35′42.4″	1919	2015/07/20
7	Portillo	S 32°50′1.4″	W 70°7′59.2″	2800	2015/07/21
8	Los Hornos	S 32°53′32.2″	W 70°12′64.1″	2322	2015/07/22
9	Valle de Maipo	S 33°29′45.8″	W 70°16′35.5″	2302	2015/07/26
10	Valle Nevado	S 33°21′58,3″	W 70°15′16.3″	2635	2015/07/24
11	La Parva	S 33°20′06″	W 70°18′00.7″	2610	2016/07/25
12	Curicó	S 35°08′10,8″	W 70°28′45,2″	1860	2016/08/19
13	Maule	S 35°59′19,7″	W 70°33′45,1″	1860	2016/08/20
14	Chillán	S 36°54′26,4″	W 71°23′59,5″	1963	2016/07/23
15	Antuco	S 37°22′04,6″	W 71°22′57,3″	1494	2016/08/23
16	Volcán Collaqui	S 37°52′35,1″	W 71°28′26,9″	1142	2016/08/24
17	Corralco	S 38°24′21.1″	W 71°33′34.3″	1601	2016/08/27
18	Volcán Llaima	S 38°31′37,8″	W 71°47′44,5″	1830	2016/08/28
19	Volcán Villarrica	S 39°23′46,9″	W 71°57′53,0″	1450	2016/08/29
20	Antillanca	S 40°47′12,5″	W 72°11′31,5″	1349	2016/07/19
21	Volcán Osorno	S 41°07′12,0″	W 72°31′47,7″	1326	2016/08/31

Although some additional samples were collected from the subsurface layer, we focused on the surface layer since prior efforts have shown consolidation of pollutants in the surface layer with snowmelt^33^. Indeed, prior efforts at the same sampling sites allowed us to verify that subsurface layers tend to be cleaner than the surface^26^. Meteorological data suggest a lack of snowfall for at least five days before the sampling at most sampling sites. At sites 3 and 4, cold fronts brought snowfall to the area immediately prior to sampling. Although in these cases the surface layer was cleaner than the subsurface layer, element concentrations were found to be comparable (of the same order).

Researches conducting sampling in the field took care to prevent sample contamination. As also explained by Rowe *et al*.^26^, a stainless steel spatula was used and samples were placed into plastic bags, in turn packed in Whirlpack bags. Styrofoam coolers were used for transportation to the laboratory. In the laboratory deionised water was used for washing tools and containers before filtering, while latex gloves and lab coats were worn during filtering. Snow samples were transferred to a glass beaker and snowmelt was thereafter vacuum-filtered, leaving the insoluble material on the filter. Stainless-steel funnels and 0.4-µm Nucleopore filters were used. Filters were afterwards placed in sterile petri dishes.

### Chemical composition

Elemental composition of the insoluble material in the snow samples was carried out using Energy dispersive X-ray fluorescence (EDXRF) spectrometry. EDXRF is a technique widely used for elemental analysis of particles on filter media^34,35^. Elemental analysis was conducted at the Harvard School of Public Health (HSPH) using an Epsilon 5 EDXRF spectrometer (PANalytical, The Netherlands). The procedure at HSPH for analysing elements on filters is described by Kang *et al*.^35^; it involves using 49 MicroMatter XRF calibration standard polycarbonate films (Micromatter Co., Vancouver, Canada) spanning from Na (atomic number 11) to Pb (atomic number 82). When intercomparing measurements of standard films, the HSPH EDXRF spectrometer has exhibited good performance: “2% errors in precision (suggesting a good reproducibility) and 4% errors in accuracy (showing a proper calibration)”^35^. In the case of the lower concentrations associated with field samples, the HSPH EDXRF spectrometer has exhibited a reproducibility within 15% for most of the elements^35^. For this study, concentrations of Al, Mg, Si, P, Ca, Ti, S, K, V, Cr, Mn, Ni, Cu, Fe, Co, Zn, Rb, Sr, Zr, Mo, Ba and Pb were determined to be significant.

Mineralogy was assessed using X-ray powder diffraction (XRD)^36^. A Siemens D-5005 x-ray diffractometer fitted with parallel beam geometry and Cu Kα radiation, was applied. XRD patterns were collected every 0.02°, from 5° to 90°. Minerals were identified at the Instituto Venezolano de Investigaciones Científicas (IVIC) from their characteristic peaks.

### Statistical analysis

Cluster analysis and principal components analysis (PCA) were used to assess element assemblages. Before applying the multivariate statistical analysis, concentrations rendered by EDXRF were log-transformed and standardized (by subtracting the mean and dividing the outcome by the standard deviation)^37^. An orthogonal method (the varimax method) was applied for factor rotation^37^. We evaluated the following cluster analysis methods: Ward, complete linkage, single linkage, weighted pair-group average, unweighted pair-group average and unweighted pair-group centroid. STATISTICA^38^ was used to conduct the calculations.

In order to detect relative contributions of anthropogenic sources, we applied Crustal enrichment factors (EF_UCC_)^5,39–41^. EF_UCC_ was taken as the ratio between the concentration of an element and the concentration an element originated from rocks and/or dust. As pointed out by Cantonati *et al*.^41^ and Veysseyre *et al*.^42^, enrichment factors within the range 0.1–10 allow discarding the significant presence of elements other than those from rocks and dust, while EF_UCC_ values higher than 10 do suggest contributions from different sources (natural and/or anthropogenic); EF_UCC_ values within the range 10–500 (moderate enrichment) indicate input sources additional to crustal material, while EF_UCC_ values higher than 500 (high enrichment) suggest pronounced anthropogenic contribution^41,42^. In this study, EF_UCC_ values were calculated for each sampling site. In addition, a second technique for estimating metal enrichments was applied. It implied normalizing the metal concentrations against an element of detrital origin^43^. Following prior efforts^44–46^, we normalized the element concentrations against Al.

### Back-trajectory computation

Air-parcel backward trajectories were applied to sampling sites where we found anthropogenic enrichment. We used the Hybrid Single-Particle Lagrangian Integrated Trajectory (HYSPLIT) model^47–49^ fed with the Global Data Assimilation System (GDAS) archive (available in a resolution of three hours and a 1° latitude-longitude)^50^. At each site we computed 36-h backward trajectories by using meteorological data over a 92-day period prior to the sampling date (see Table 1). Over that period, trajectories were computed every 3 hours, making a total of 736 trajectories per site. These trajectories were divided into clusters following a cluster analysis based on the total spatial variance (TSV)^51^.

## Results

### Chemical composition

**Figure 2:**
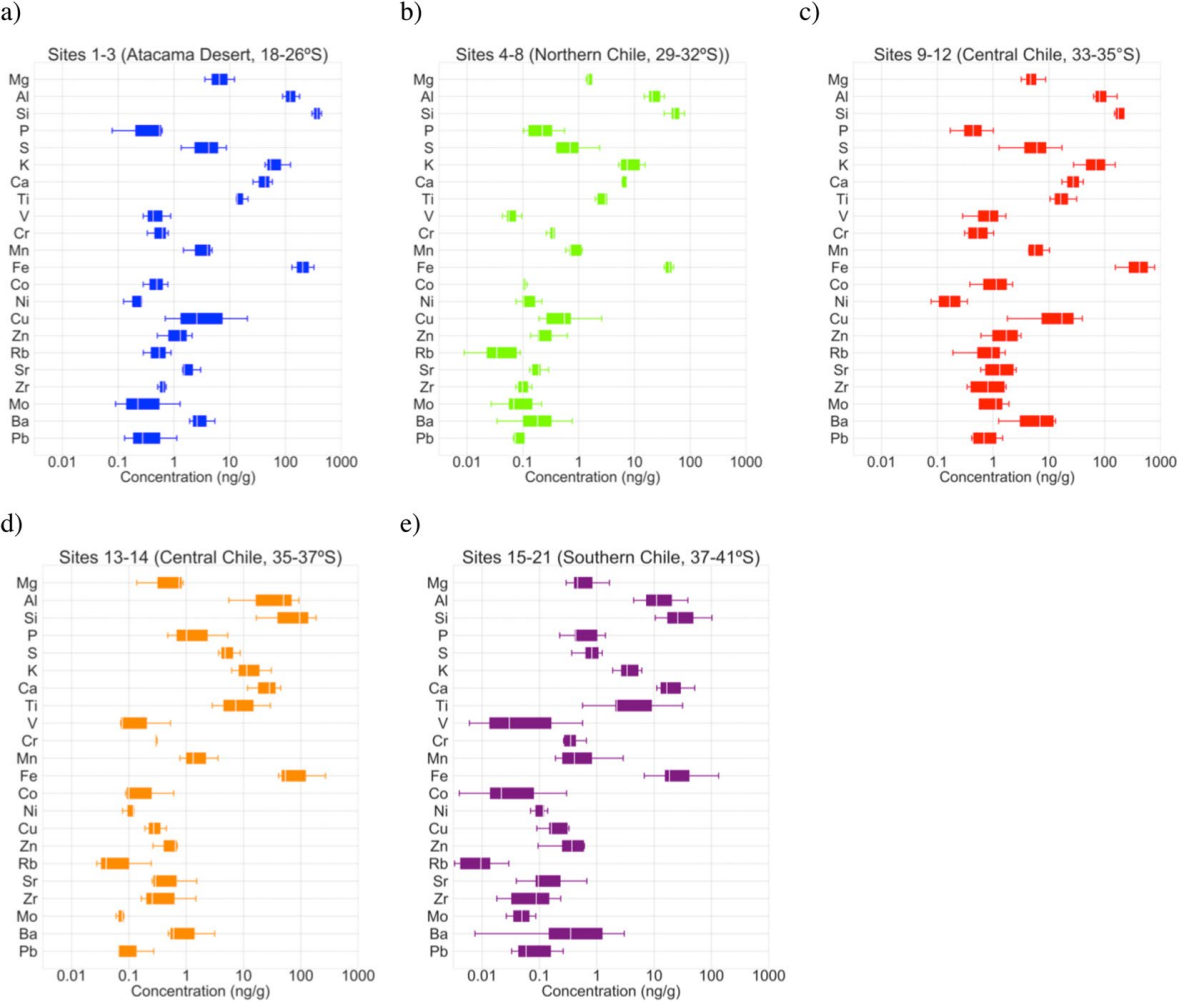
Element concentrations (ng of the element per g of snow) in the snow samples collected in: (**a**) sites 1–3 (in the Atacama Desert); (**b**) sites 4–8 (in northern Chile); (**c**) site 9–12 (in central Chile); (**d**) sites 13 and 14 (also in central Chile); and e) sites 15–21 (in southern Chile). In each box, the central mark (white stripe) indicates the median, and the edges indicate the 25th and 75th percentiles. The whiskers extend to the most extreme data points. Plots generated by using PYTHON’s Matplotlib Library (https://matplotlib.org)^62^.

Figure 2 shows the boxplots of the element concentrations (ng of the element per g of snow) in the snow samples collected in the five depositional environments indicated in Fig. 1. As shown in Fig. 2, for all sets, either Si or Fe exhibited the highest concentration followed in general by K or Ca, Al, Ti and Mg. Figure 2 also allows realizing the dispersion of element concentrations found in Andean snow (18–41°S). It can be observed that the concentrations of Co, Cu, Rb, Ba and V ranged within three orders of magnitude. Concentrations of Mg, P, K, Ni, Zn, Sr, Zr, Ti, Mn, Mo, Pb and Fe ranged within two orders of magnitude, while the other elements ranged only within one order of magnitude.

Table 2 shows the best estimates of the element concentrations (ng of element per g of snow) in the snow samples at each site while the associated uncertainty are shown in Table 3. We found relatively high concentrations of K, Ti, V, Mn, Fe, Co, Zn, Rb, Sr, Ni, Cu, Zr, Mo, Ba and Pb at site 11 (La Parva, nearby Santiago), while at site 2 (Cerro Toco, in the Atacama Desert) relatively high concentrations of Al and Si were detected.

**Table 2:** Best estimates of the element concentrations (ng of the element per g of snow) in the snow sampled at each site.

Sites	Mg (ng/g)	Al (ng/g)	Si (ng/g)	P (ng/g)	S (ng/g)	K (ng/g)	Ca (ng/g)	Ti (ng/g)	V (ng/g)	Cr (ng/g)	Mn (ng/g)	Fe (ng/g)	Co (ng/g)	Ni (ng/g)	Cu (ng/g)	Zn (ng/g)	Rb (ng/g)	Sr (ng/g)	Zr (ng/g)	Mo (ng/g)	Ba (ng/g)	Pb (ng/g)
1	3.587	121.341	367.877	0.616	4.222	53.901	26.146	13.198	0.281	0.791	1.477	130.249	0.281	0.268	0.698	0.502	0.282	1.435	0.511	0.226	2.598	0.130
2	6.541	178.911	442.749	0.078	8.712	122.832	43.788	21.269	0.873	0.624	4.829	323.442	0.778	0.265	20.713	2.114	0.886	3.014	0.725	1.291	5.425	1.125
3	12.186	88.691	298.604	0.551	1.345	43.023	58.090	13.605	0.424	0.333	3.989	203.550	0.493	0.125	2.567	1.304	0.547	1.524	0.641	0.090	1.911	0.275
4	3.678	34.431	78.880	0.129	0.981	12.261	7.397	3.032	0.074	0.316	1.170	50.449	0.102	0.104	0.195	0.190	0.077	0.193	0.122	0.057	0.184	0.106
5	1.504	18.711	47.340	0.329	0.402	5.853	7.383	2.223	0.053	0.351	0.700	36.857	0.109	0.100	0.271	0.193	0.023	0.133	0.085	0.027	0.104	0.074
6	1.718	28.409	62.647	0.224	2.378	15.621	6.031	3.182	0.096	2.008	0.585	45.551	0.120	0.163	2.587	0.322	0.090	0.290	0.098	0.216	0.321	0.068
7	1.359	20.800	51.656	0.560	0.705	7.435	21.109	3.187	0.043	0.369	1.104	42.290	0.105	0.220	0.713	0.632	0.034	0.204	0.146	0.145	0.766	0.106
8	1.440	15.049	34.085	0.102	0.406	5.203	6.129	1.975	0.056	0.263	0.715	34.105	0.065	0.076	0.557	0.138	0.009	0.150	0.074	0.069	0.034	0.071
9	5.078	89.933	186.581	0.376	7.043	86.686	17.095	13.960	0.684	0.387	4.408	334.266	0.822	0.122	12.187	1.161	0.747	0.793	0.425	0.969	4.151	0.462
10	3.185	63.031	149.849	0.171	5.277	56.360	31.794	18.978	1.082	1.023	6.975	526.017	1.584	0.228	23.916	2.571	1.195	2.178	1.529	1.300	11.494	1.020
11	8.746	167.006	369.742	1.009	17.154	154.360	41.285	31.442	1.694	0.714	10.258	785.067	2.237	0.347	39.692	3.168	1.652	2.592	1.713	1.920	13.154	1.484
12	4.323	72.167	165.077	0.516	1.275	27.685	23.528	10.489	0.284	0.308	4.345	154.625	0.383	0.077	1.800	0.605	0.190	0.599	0.342	0.112	1.258	0.413
13	0.138	5.558	16.731	5.322	3.660	6.217	28.410	2.844	0.072	0.311	1.322	40.958	0.088	0.078	0.271	0.647	0.040	0.254	0.165	0.076	0.602	0.066
14	0.754	49.986	95.366	0.476	4.780	11.392	11.753	7.308	0.077	0.289	0.778	53.881	0.100	0.123	0.191	0.264	0.028	0.292	0.257	0.060	0.487	0.066
15	0.421	11.026	25.893	0.733	0.805	2.874	11.134	2.184	0.030	0.658	0.417	18.547	0.036	0.119	0.328	0.361	0.011	0.095	0.088	0.026	0.008	0.058
16	0.795	8.424	18.684	0.424	1.247	4.536	11.643	31.491	0.566	0.460	0.190	20.870	0.020	0.139	0.996	25.280	0.003	0.111	0.018	0.289	1.658	0.260
17	0.398	38.936	84.424	1.373	0.940	6.017	51.213	11.262	0.140	0.264	2.896	133.949	0.297	0.070	0.281	0.609	0.014	0.672	0.236	0.035	0.508	0.178
18	0.852	11.077	26.759	0.431	0.820	3.380	15.112	2.247	0.023	0.282	0.408	17.513	0.022	0.117	0.161	0.564	0.003	0.096	0.113	0.050	0.127	0.135
19	0.292	6.523	16.174	1.418	1.175	2.570	19.116	2.130	0.006	0.351	0.327	14.209	0.010	0.117	0.145	0.369	0.000	0.081	0.039	0.086	0.242	0.033
20	0.462	4.405	10.494	0.226	0.522	1.897	16.705	0.566	0.008	0.269	0.199	6.740	0.004	0.077	0.090	0.095	0.008	0.040	0.028	0.049	0.000	0.046
21	1.665	36.423	102.150	0.420	0.365	6.108	43.026	7.129	0.179	0.392	1.576	78.890	0.170	0.098	0.157	0.177	0.029	0.473	0.186	0.037	3.022	0.042

**Table 3:** Uncertainty associated with the element concentrations shown in Table 2.

Sites	u(Mg) (ng/g)	u(Al) (ng/g)	u(Si) (ng/g)	u(P) (ng/g)	u(S) (ng/g)	u(K) (ng/g)	u(Ca) (ng/g)	u(Ti) (ng/g)	u(V) (ng/g)	u(Cr) (ng/g)	u(Mn) (ng/g)	u(Fe) (ng/g)	u(Co) (ng/g)	u(Ni) (ng/g)	u(Cu) (ng/g)	u(Zn) (ng/g)	u(Rb) (ng/g)	u(Sr) (ng/g)	u(Zr) (ng/g)	u(Mo) (ng/g)	u(Ba) (ng/g)	u(Pb) (ng/g)
1	0.290	0.777	1.425	0.016	0.025	0.014	0.101	0.099	0.041	0.006	0.012	0.030	0.422	0.009	0.010	0.033	0.017	0.014	0.025	0.037	0.040	0.299
2	0.267	1.021	1.696	0.010	0.035	0.010	0.204	0.149	0.057	0.005	0.009	0.034	0.987	0.008	0.007	0.076	0.019	0.013	0.026	0.029	0.035	0.258
3	0.248	0.505	1.138	0.007	0.008	0.006	0.073	0.186	0.035	0.003	0.004	0.022	0.617	0.005	0.004	0.018	0.010	0.007	0.013	0.015	0.015	0.119
4	0.133	0.232	0.311	0.005	0.007	0.004	0.025	0.029	0.011	0.002	0.004	0.012	0.162	0.003	0.003	0.011	0.006	0.005	0.008	0.012	0.014	0.097
5	0.115	0.161	0.195	0.007	0.006	0.005	0.016	0.030	0.009	0.002	0.005	0.012	0.123	0.004	0.004	0.013	0.007	0.005	0.008	0.014	0.016	0.108
6	0.079	0.182	0.245	0.004	0.010	0.003	0.028	0.023	0.010	0.001	0.006	0.008	0.143	0.002	0.003	0.014	0.005	0.003	0.006	0.008	0.010	0.067
7	0.116	0.173	0.211	0.008	0.007	0.006	0.018	0.073	0.011	0.002	0.005	0.014	0.140	0.004	0.004	0.014	0.008	0.005	0.009	0.014	0.017	0.118
8	0.088	0.124	0.140	0.004	0.005	0.004	0.013	0.024	0.007	0.002	0.003	0.009	0.111	0.003	0.003	0.010	0.005	0.004	0.006	0.010	0.011	0.078
9	0.142	0.502	0.715	0.006	0.025	0.004	0.140	0.058	0.036	0.003	0.004	0.022	1.004	0.005	0.003	0.041	0.009	0.007	0.009	0.013	0.016	0.126
10	0.112	0.364	0.576	0.005	0.019	0.004	0.093	0.103	0.047	0.003	0.005	0.029	1.572	0.007	0.003	0.070	0.014	0.009	0.014	0.017	0.017	0.195
11	0.253	0.931	1.414	0.012	0.058	0.007	0.250	0.138	0.079	0.006	0.007	0.047	2.352	0.012	0.006	0.118	0.021	0.014	0.021	0.028	0.031	0.288
12	0.140	0.419	0.635	0.007	0.008	0.005	0.049	0.079	0.028	0.002	0.004	0.022	0.471	0.004	0.003	0.015	0.007	0.005	0.009	0.013	0.014	0.106
13	0.085	0.087	0.077	0.030	0.015	0.005	0.015	0.094	0.010	0.002	0.004	0.013	0.134	0.003	0.003	0.011	0.008	0.005	0.008	0.012	0.014	0.099
14	0.093	0.308	0.373	0.007	0.018	0.006	0.023	0.043	0.021	0.002	0.004	0.011	0.172	0.003	0.003	0.011	0.006	0.005	0.008	0.013	0.014	0.098
15	0.089	0.114	0.112	0.008	0.007	0.005	0.010	0.041	0.009	0.002	0.005	0.010	0.067	0.003	0.003	0.012	0.006	0.004	0.007	0.012	0.014	0.093
16	0.092	0.099	0.084	0.006	0.008	0.006	0.013	0.042	0.077	0.003	0.004	0.009	0.074	0.003	0.003	0.013	0.104	0.004	0.007	0.011	0.014	0.108
17	0.086	0.252	0.331	0.011	0.007	0.006	0.015	0.164	0.030	0.002	0.004	0.017	0.409	0.004	0.003	0.011	0.007	0.004	0.009	0.012	0.013	0.096
18	0.090	0.111	0.114	0.006	0.006	0.006	0.011	0.052	0.008	0.002	0.004	0.009	0.063	0.003	0.003	0.010	0.007	0.004	0.007	0.012	0.013	0.089
19	0.087	0.091	0.075	0.011	0.008	0.005	0.010	0.065	0.008	0.002	0.004	0.010	0.054	0.003	0.003	0.011	0.007	0.004	0.007	0.012	0.014	0.096
20	0.087	0.079	0.054	0.005	0.006	0.005	0.008	0.058	0.005	0.002	0.004	0.009	0.032	0.003	0.003	0.011	0.005	0.004	0.007	0.012	0.013	0.091
21	0.120	0.251	0.401	0.007	0.006	0.007	0.016	0.140	0.021	0.002	0.005	0.015	0.248	0.004	0.004	0.013	0.007	0.005	0.010	0.015	0.016	0.140

We found relatively high concentrations of Mg and Ca at site 3 (La Ola, in northern Chile), whereas at site 10 (Valle Nevado, also near Santiago), 14 (Chillán, in central Chile) and 19 (Volcán Villarrica, in southern Chile), relatively high concentrations of Cr, S and P, were measured, respectively.

The lowest concentrations of Si, K, Ti, Fe, Co, Ni, Cu, Zn, Sr, Zr and Ba were found at site 20 (Antillanca, in southern Chile), while at site 19 (Volcán Villarrica, also in southern Chile) we detected the lowest concentrations of V, Rb and Pb.

The lowest concentrations of Al and Mo were measured at site 15 (Antuco, central Chile), while at sites 2 (Cerro Toco, in the Atacama Desert), 6 (Valle Choapa, in northern Chile), 8 (los Hornos, in northern Chile), 13 (Maule, in central Chile), 16 (Volcán Collaqui, in southern Chile) and 21 (Volcán Osorno, also in southern Chile), we found the lowest concentrations of P, Ca, Cr, Mg, Mn and S, respectively. See Table 2 for further details.

### Statistical analysis

Cluster analysis and PCA were carried out on the dataset in Table 2 (after normalization and standardization). Table S1 shows the correlation matrix for the element concentrations.

**Figure 3:**
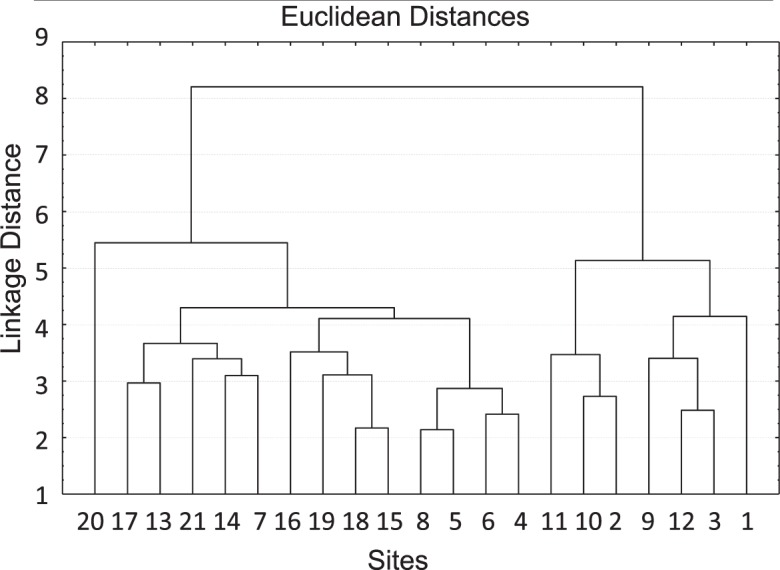
Dendrogram obtained from the cluster analysis of elemental concentrations using the unweighted pair-group average method. Numbers in the horizontal axis identify the 21 sampling sites indicated in Table 1. Plot generated by using STATISTICA (data analysis software system, version 7, http://www.statsoft.com/Products/STATISTICA-Features)^38^.

Figure 3 shows the dendrogram obtained from the cluster analysis (of elemental concentrations) using the unweighted pair-group average method. Two groups are identified: (i) group 1 consists of sites 1–3 (in the northern Atacama Desert, 18–26°S), sites 9–11 (relatively close to Santiago, ~33°S) and site 12 (close to Curicó, ~35°S); (ii) group 2 consists of sites 4–8 (in northern Chile, 29–32°S), sites 13–14 (in central Chile, 35–37°S), and sites 15–21 (in southern Chile, 37–41°S). Dendograms from the cluster analysis for other linkage methods (the single linkage method, complete linkage method, unweighted pair-group centroid method, Ward’s method, and weighted pair-group average method) consistently suggest the same two groups shown in Fig. 3.

**Figure 4:**
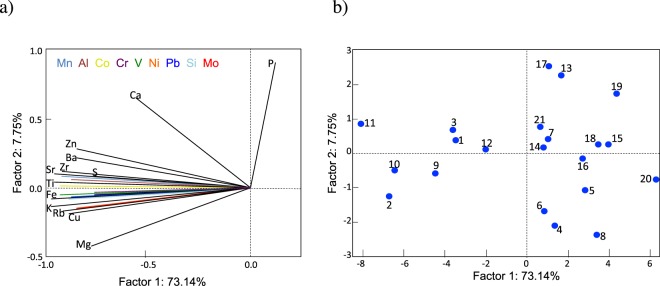
Principal component analysis (PCA). Plots generated by using STATISTICA (data analysis software system, version 7, http://www.statsoft.com/Products/STATISTICA-Features)^38^. (**a**) Projections of elements on the factor-plane. (**b**) Projections of sites on the factor-plane; numbers identify the 21 sampling sites indicated in Table 1.

PCA results are shown in Table 4, while Fig. 4 shows scores and loading. Two principal components accounted for 81% of the total variance. Component 1 (eigenvalue equal to 16.1) presented significant negative loads of Mg, Al, Si, S, K, Ti, V, Cr, Mn, Fe, Co, Ni, Cu, Zn, Rb, Sr, Zr, Mo, Ba and Pb. Component 2 (eigenvalue equal to 1.7) has a high positive load of P. The PCA results (see Fig. 4b) also distinguished the same two groups suggested by the cluster analysis.

**Table 4:** Principal factors obtained after a varimax rotation.

Element	Factor 1 (73%)	Factor 2 (8%)
Mg	**−0**.**78157**	−0.410716
Al	**−0**.**88805**	0.064792
Si	**−0**.**90495**	−0.069823
P	0.11635	**0**.**906353**
S	**−0**.**76154**	0.088118
K	**−0**.**97397**	−0.132094
Ca	−0.56330	0.651318
Ti	**−0**.**95559**	0.045982
V	**−0**.**93353**	−0.047603
Cr	**−0**.**76222**	−0.025786
Mn	**−0**.**92033**	0.080591
Fe	**−0**.**97410**	−0.074116
Co	**−0**.**92930**	0.014289
Ni	**−0**.**76986**	−0.044991
Cu	**−0**.**88857**	−0.179315
Zn	**−0**.**84932**	0.285502
Rb	**−0**.**92863**	−0.156184
Sr	**−0**.**96084**	0.103885
Zr	**−0**.**92083**	0.126097
Mo	**−0**.**86036**	−0.141991
Ba	**−0**.**85242**	0.220138
Pb	**−0**.**88566**	−0.060095

The percentages of each factor are shown in parentheses. Two factors explain 81% of the total variance.

### Mineralogy

Table 5 shows the bulk mineralogy of the snow samples, as the percentage of each mineral in the insoluble material filtered from snow. The main mineralogical species were: silicates (labradiorite, microcline, albite), hydrated silicates (muscovite, laumontite, K-Fe-Mg-Al hydroxysilicate, biotite), quartz, hydrated sulphates, oxides, hydrated oxides, as well as hydrated arsenates at sites 15–21 (in southern Chile).

**Table 5:** Bulk mineralogy of the snow samples.

Site	qz	sil	h.sil	oxi	h.oxi	h.sulp	h.pho	fluo	h.arse	carb	h.carb	rar.er	unin.ph
1	***	*****	*****	**		***							
2	****	******	***	***		*							
3	***	******	***	***		***							
4	**	***	*****	**		***	***						
5	***	*****	****	*		***	**						
6	**	****	******	***		***							
7	***	*****	****	****		*							
8	***	*****	****	*	**	***							
9	**	***	******	*	*	**							
10	***		****	**	*	******							
11	*****	*	***	**	*	***	***						
12	****	*	******	**			**	***					
13	***	****	***		***	****							
14	***	*****	*****	**		*	**				*		
15		*****				****			*****				
16		*****	****	**			****		**				
17			****	**	***		***		*****			**	
18		****	***						****		***		
19			*****	**	****	***		**	***				
20		*****	**			***			***	***	***		
21		******	**			***			***			**	**

qz: quartz, sil: silicates, h.sil: hydrated silicates, oxi: oxides, h.oxi: hydrated oxides, h.sulp: hydrated sulphates, h.pho: hydrated phosphates, fluo: fluorides, h.arse: hydrated aresenates, carb: carbonates, h.carb: hydrated carbonates, rar.er: rare earth, and unin.ph: unidentified phases.

* < 5%, **(5–10)%, ***(11–20)%, ****(21–30)%, *****(31–40)%, ****** > 40%.

### Enrichment factors

Table 6 shows the enrichment factors (EF_UCC_) of elements found in the snow. Mg, Si, P, S, K, Ca, Ti, Sr and Zr showed no significant enrichment (EF_UCC_ < 10) in the snow samples. In the case of V, Cr, Mn, Fe, Co, Ni, Zn, Rb, Ba and Pb, EF_UCC_ distributions ranged from no enrichment at some sites to moderate enrichment (10 < EF_UCC_ < 500) at others. Cu and Mo presented in general relatively high enrichments, from moderate (10 < EF_UCC_ < 500) to intense (EF_UCC_ > 500). Our results show a high enrichment of Cu at site 2 (Cerro Toco, in the Atacama Desert) and both Cu and Mo at sites 9–11 (close to Santiago).

**Table 6:** Crustal enrichment factor (EF_UCC_).

Sites	Mg	Si	P	S	K	Ca	Ti	V	Cr	Mn	Fe	Co	Ni	Cu	Zn	Rb	Sr	Zr	Mo	Ba	Pb
1	0.2	1	1	3	1	1	3	3	14	2	3	15	9	31	6	2	3	1	103	2	5
2	0.2	1	0.1	4	2	1	3	7	8	4	5	29	6	**627**	18	3	4	1	399	4	29
3	0.5	1	1	1	1	2	4	7	8	7	6	37	6	157	22	4	4	2	56	2	14
4	1	1	0.4	2	1	1	2	3	20	5	4	20	13	31	8	2	1	1	92	1	14
5	0.5	1	2	2	1	1	3	4	41	5	5	39	22	78	15	1	2	1	80	1	18
6	0.3	1	1	7	1	1	3	5	26	3	4	28	15	493	17	2	2	1	421	1	11
7	0.4	1	3	3	1	3	4	3	39	8	5	34	24	186	45	1	2	2	387	4	23
8	0.5	1	1	2	1	1	3	5	39	7	6	29	21	201	14	0.4	2	2	253	0.3	22
9	0.3	1	0.5	6	3	0	4	11	10	7	9	61	6	**734**	19	6	2	2	**596**	5	23
10	0.3	1	0.3	7	2	1	7	25	36	16	21	168	15	**2055**	61	13	8	8	**1141**	21	74
11	0.3	1	1	3	2	1	5	15	9	9	12	89	9	**1287**	28	7	4	3	**636**	9	40
12	0.3	1	1	1	1	1	4	6	9	9	5	35	4	135	12	2	2	2	86	2	26
13	0.1	0.2	6	12	1	3	3	4	27	8	4	23	13	57	38	1	2	2	165	3	12
14	0.1	0.5	1	8	1	1	4	2	13	2	3	13	10	21	8	0.4	1	2	66	1	6
15	0.2	1	8	6	1	3	5	4	72	6	4	22	42	161	49	1	2	3	132	0.2	24
16	0.2	0.2	2	4	0.4	1	3	9	28	1	2	5	16	190	20	0.1	1	0.2	95	7	26
17	0.1	1	4	2	0.4	3	1	5	15	6	2	51	7	39	23	0.3	4	2	50	2	21
18	0.4	1	5	6	1	4	5	3	56	5	4	13	40	79	76	0.2	2	3	249	1	56
19	0.1	0.2	6	4	0.3	2	2	0.3	29	2	1	2	18	30	21	0.1	1	0.5	180	1	6
20	0.1	0.1	1	2	0.2	2	1	0.5	24	1	1	1	13	20	6	0.2	0.4	0.4	112	0.2	9
21	0.3	1	1	1	0.5	3	5	7	24	6	5	31	11	23	7	1	3	2	56	10	5

Figures S1–S5 show the correlation between Al concentration and the concentration of Ti, V, Cr, Mn, Fe, Co, Ni, Cu, Zn, Rb, Sr, Zr, Mo, Ba and Pb. The dashed lines in Figs S1–S5 represent 95% confidence interval. Values found well above these lines represent metals enrichment at a particular site. Again, our results indicate enrichment of Ni, Mn, Ti, V, Co, Cu, Zn, Zr, Ba and Pb at site 10 (Valle Nevado) and at site 11 (La Parva); of Fe, Rb and Mo at sites 9–11 (Valle de Maipo, Valle Nevado, and La Parva); and of Sr and Cr at site 10 (Valle Nevado). These results are consistent with significant enrichments previously reported^14^ in the case of snow sampled at Cerro Colorado (in the very same area of our sampling sites 10 and 11). The correlations in Figs S1–S5 also suggest enrichments of Cr and Ni at site 1 (Putre in the Atacama Desert), and of Mn at site 12 (close to Curicó).

## Discussion

### Possible Sources

As pointed out above, cluster analysis and PCA of the element concentrations in the snow samples suggested two main groups: i) group 1 consists of sites 1–3 (18–26°S) and sites 9–12 (33–35°S); ii) group 2 consists of sites 4–8 (29–32°S), sites 13–14 (35–37°S), and sites 15–21 (37–41°S). Group 1 (sites 1–3 and 9–12) exhibited significantly higher concentrations of Mg, Al, Si, S, K, Ti, V, Cr, Mn, Fe, Co, Ni, Cu, Zn, Rb, Sr, Zr, Mo, Ba and Pb than group 2 (sites 4–8 and 13–21). No significant difference was found for P. Except for Cu and Mo (see details below), the metal concentrations found in snow samples of group 2 are comparable to metal concentrations (attributed to deposition of background atmospheric aerosols) previously reported for snow samples collected in remote areas in Europe (Eastern Alps^2^, Central Pyrenees^5^, and French Alps^42^), and North America (Alaska^6^).

The two principal components (that explained 81% of the total variance in the PCA) suggest the origin of the elements in each group. Component 1 is characterized by high negative loads of Mg, Al, Si, S, K, Ti, V, Cr, Mn, Fe, Co, Ni, Cu, Zn, Rb, Sr, Zr, Mo, Ba and Pb, while Component 2 exhibits a high positive load of P. Component 1 includes elements that are abundant in the crust (Al, Ti, Mn and Fe), which pointed at terrigenous dust deposition^5^. Moreover, Component 2 includes P, often used in herbicides and pesticides. The position of sites 13 (Maule) and 17 (Corralco) in Fig. 4b highlights the loading of P in snow samples at these sites. However, the low crustal enrichment factor for P (see Table 6) does not support significant effects of the agricultural activity on the snowpack in the area of Maule and Corralco.

In all of the samples, Cu and Mo exhibited the highest enrichment factors (EF_UCC_), from moderate to intense. Element concentrations of both Cu and Mo show a very good correlation (Pearson coefficient = 0.98, see Table S1), which suggests that they have a common origin. This result is consistent with the fact that concentrations of hypogene Cu (0.40–0.86%) and Mo (0.01–0.025%) have been reported for the Andes in 18 major deposits (in Chile, Argentina and southern Peru)^52^. Chile is currently the top producer of copper (accounting for about one third of the global copper production) and ranked third in molybdenum production^28^.

**Figure 5:**
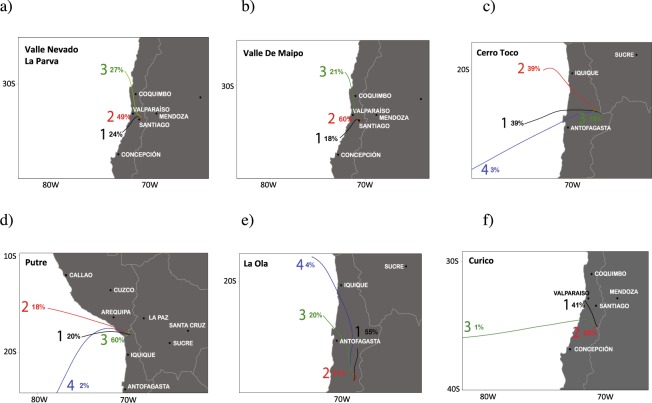
36-h back trajectory clusters. (**a**) Valle Nevado/La Parva; (**b**) Valle del Maipo; (**c**) Cerro Toco; (**d**) Putre; (**e**) La Ola; (**f**) Curicó. Plots generated by the using MeteoInfo software tools for meteorological data visualization^63^.

Figure 5 shows the 36-h back trajectory clusters for sites where we found significant anthropogenic enrichment. Back trajectories were computed every 3 hours using meteorological data over a 92-day period prior to the sampling date making a total of 736 trajectories for each site. Back trajectories from sites 9–12 were divided into 3 clusters, while trajectories from sites 1–3 were divided into 4 clusters.

Figure 5a,b show the back trajectory clusters for sites 9–11, which are situated less than 20 km east of heavily populated urban areas of Santiago (clusters for Valle Nevado and La Parva are shown in a single plot since these sites are very close each other). Cluster 2 was the largest in Fig. 5a,b. This cluster suggests a heavy influence from Santiago, as a majority of the trajectories (49% in Fig. 5a and 60% in Fig. 5b) originated in or passed over Santiago, and are characterized by being short, with low wind speeds. The second most frequent cluster in Fig. 5a (Fig. 5b) was number three, which contained 27% (21%) of all trajectories. These trajectories typically indicated a source area in northern Chile but passing close to Los Bronces (a major copper mine). Sites 9 (Valle del Maipo), 10 (Valle Nevado) and 11 (La Parva) are located about 30 km south in the case of site 9 (and about 15 km south in the case of sites 10 and 11) of Los Bronces. Cu and Mo exhibited high enrichment factors (EF_UCC_ > 500) at sites 9–11.

Cu also exhibited a high enrichment factor (EF_UCC_ > 500) at site 2 (Cerro Toco). This site is located in the Atacama Desert, a region whose main economic activity is the copper mining industry. Figure 5c shows that Cluster 2 contained 39% of the trajectories for Cerro Toco. This cluster represented air coming out of the northwest passing close to several major copper mines (Radomiro Tomic, Ministro Hales and Chuquicamata, the latter being one of the largest copper mines in the world). Cerro Toco is located within 150 km of these major copper mines. Cluster 1 also contained 39% of all trajectories for Cerrro Toco. These trajectories typically indicated a source area west of Cerro Toco and pass over important astronomical projects (powered by diesel generators) located only few km west of the sampling site.

Figure 5d,e shows the back trajectory clusters for site 1 (Putre) and site 3 (la Ola). Cluster 3 in Fig. 5d, containing 60% of all trajectories, was the largest. The routes for this cluster were typically short and indicated a slow wind speed. The majority of these trajectories originated in the area close to Tacna (a Peruvian city of about 300,000 inhabitants) and to a major gold mine (Pucamarca) in the Peruvian side of the border. Site 1 is located about 70 km east of Tacna and about 40 km southeast of the Pucamarca mine. In the case of site 3 (La Ola), Fig. 5e shows that Cluster 1 contained 55% of the trajectories. This cluster represented air coming out of the north. Some trajectories in this cluster originated from nearby a major copper mine (La Escondida), located about 220 km north of the sampling site. The second most frequent cluster in the case of La Ola was number two, which contained 21% of all trajectories. These trajectories typically indicated a source area about 60 km northwest of the sampling site, close to another major copper mine (El Salvador).

Figure 5f shows the clusters for site 12 (Curicó). Cluster 2 was the largest of the 3, containing 58% of the back trajectories. These trajectories were typically characterized by slow air movement, with the trajectories extending about 60 km from the west to the receptor site. Cluster 1 was the second most common path. This cluster contained 41% of the total trajectories. These trajectories were faster and pass over the most populated and industrialized areas in Chile. Still, due to the moderate EF_UCC_ values, an anthropogenic influence can be considered modest at site 12.

### Volcanic activity

There are more than one hundred volcanoes in the Chilean Andes that have been active during the Holocene^31^. Several of these volcanoes are currently active^32^. Areas surrounding active volcanoes are subjected to volcanic ash deposition^30^. In active geothermal systems in the southern Andes of Chile, As is present in borehole fluids^53^. In thermal waters in the southern volcanic zone (SVZ), As is transported from geothermal reservoirs to the surface^54^. In eruptive gases, arsenic predominates in the form of As(OH)_3_^55^. Ash can absorb As(OH)_3_ (as well as similar to volatile compounds)^56^. A part of the magmatic arsenic could also be incorporated into the glass structure^57,58^. As discussed by Bia *et al*.^58^, partial remobilization of structural As can occur when dissolution of aluminosilicate glass dissolute, which is favored by extreme acid conditions; volatile acids (such as HCl, H_2_S, and HF) in the volcanic plume can produce such conditions^58^.

Silicates (quartz, silicates and hydrated silicates in Table 5) were found to be the dominant minerals at every sampling site. However, the volcanic activity fingerprint on the snow was apparent in samples taken in southern Chile. Our results indicate that sites 15–21 have relatively low quartz concentration and high concentrations of arsenates. These sites are located in the very active SVZ, where prior studies have shown absence^59^ or minor amount^60^ of quartz in ashes. The predominant minerals detected in fault-controlled hydrothermal systems in the southern Andes are Ca and Ca (-Na) zeolites, minor quartz was also locally found^61^. The relatively low quartz concentration at sites 15–21 suggests a reduction of amorphous silica by carbon monoxide during the frequent explosive eruptions in the zone.

## Summary and Conclusions

We have carried out a chemical composition analysis (major and trace elements, mineralogy, and chemical enrichment) of surface snow sampled across a transect of about 2,500 km in the Chilean Andes (18–41°S).

Cluster analysis and principal component analysis (PCA) applied to the element concentration data, rendered consistent figures suggesting two main groups: (i) group 1 consists of sites 1–3 (in the Atacama Desert, 18–26°S), sites 9–11 (close to Santiago, ~33°S) and site 12 (close to Curicó, ~35°S); (ii) group 2 consists of sites 4–8 (in northern Chile, 29–32°S), sites 13–14 (in central Chile, 35–37°S), and sites 15–21 (in southern Chile, 37–41°S).

Group 1 exhibited significantly higher concentrations of several metals (Al, Si, Ti, Cr, Fe, Co, Ni, Cu, Zn, and Pb) than group 2. Indeed, with the exception of Cu and Mo, metal concentrations found in snow samples of group 2 are comparable to metal concentrations (attributed to the deposition of background atmospheric aerosols) previously reported for snow samples collected in remote areas elsewhere.

Cu and Mo exhibited the highest enrichment factors (moderate-intense) in all samples. The very good correlation between concentrations of both Cu and Mo is consistent with the fact that major mineral deposits in the Andes contain both elements.

Our results confirm significant anthropogenic enrichment (Cu and Mo) at sites near Santiago (sites 9–11). These sites are located about 30 km south in the case of site 9 (and about 15 km south in the case of sites 10 and 11) of Los Bronces, a major copper mine. Anthropogenic enrichment (Cu) was also found at Cerro Toco in the Atacama Desert (site 2), located within 150 km of several major copper mines (Radomiro Tomic, Ministro Hales and Chuquicamata).

Moreover, our results confirm the influence of Santiago (a major city of 6 million inhabitants) on the snowpack nearby. We found significant enrichment of Ni, Mn, Ti, V, Co, Cu, Zn, Zr, Ba and Pb at site 10 (Valle Nevado) and at site 11 (La Parva); of Fe, Rb and Mo at sites 9–11 (Valle de Maipo, Valle Nevado, and La Parva); and of Sr and Cr at site 10 (Valle Nevado). These results were expected since these sites are located less than 20 km east of heavily populated urban areas of Santiago.

We have also detected the fingerprint of volcanic ash deposition on snow samples taken in southern Chile (37–41°S), an area with a dozen of active volcanoes. Our results at sites 15–21 exhibited a relatively low quartz concentration and high concentrations of arsenates, which is consistent with prior studies that showed the absence or minor amounts of quartz in ashes.

Our elemental and mineralogical analysis allowed us to identify five depositional environments: (i) sites 1–3 (in the Atacama Desert, 18–26°S) with relatively high concentrations of metals, high abundance of quartz and low presence of arsenates, (ii) sites 4–8 (in northern Chile, 29–32°S) with relatively high abundance of quartz and low presence of metals and arsenates, (iii) sites 9–12 (in central Chile, 33–35°S) with anthropogenic enrichment of metals, relatively high abundance of quartz and low presence of arsenates, (iv) sites 13–14 (also in central Chile, 35–37°S) with relatively high values of quartz and low abundance of metals and arsenates, and v) sites 15–21 (in southern Chile, 37–41°S) with relatively high presence of arsenates and low abundance of metals and quartz.

Our results have important implications for many Andean communities, especially in central Chile (33–37°S), where we found significant anthropogenic enrichment. In this region the snowpack is important for urban water supply, power generation, and agriculture^22^. Moreover, our findings are consistent with prior efforts^26^ that have shown that presence of higher concentrations of light-absorbing impurities in the Andean snow in the Atacama Desert and at locations nearby Santiago.

## Supplementary information

Supplementary Information

## Data Availability

The datasets generated and analyzed during the current study are available from the corresponding author on reasonable request. For the trajectory analysis, we used the Global Data Assimilation System (GDAS1) Archive Information provided through the Real-time Environmental Applications and Display sYstem (READY) (http://ready.arl.noaa.gov/gdas1.php).
