# Supplementary material for: Elemental and Mineralogical Composition of the Western Andean Snow (18°S–41°S)
Source: Sci Rep. 2019 May 31;9:8130. doi: 10.1038/s41598-019-44516-5 (PMC6544652; doi:10.1038/s41598-019-44516-5)
Supplement: Supplementary file 1 — Supplementary Information [file 41598_2019_44516_MOESM1_ESM.pdf]

Supplementary information

Elemental and Mineralogical Composition of the Western Andean Snow (18ºS -41ºS)

Juan A. Alfonso[1,2], Raul R. Cordero[1,*], Penny M. Rowe[1,3], Steven Neshyba[4], Gino Casassa[5,6], Jorge Carrasco[6], Shelley MacDonell[7], Fabrice Lambert[8,9], Jaime Pizarro[1], Francisco Fernandoy[10], Sarah Feron[1,11], Alessandro Damiani[1,12], Pedro Llanillo[1], Edgardo Sepulveda[1], Jose Jorquera[1], Belkis Garcia[2], Juan M. Carrera[2], Pedro Oyola[13], Choong-Min Kang[14]

1. Universidad de Santiago, Av. B. O'Higgins 3363, Estación Central, Chile
2. Instituto Venezolano de Investigaciones Científicas (IVIC), Apartado 20632, Caracas 20632, Venezuela
3. NorthWest Research Associates, Redmond, USA
4. Dept. of Chemistry, University of Puget Sound, Tacoma, USA
5. Unidad de Glaciología y Nieves, Ministerio de Obras Públicas, Chile
6. Centro GAIA Antártica, Universidad de Magallanes, Punta Arenas, Chile
7. Centro de Estudios Avanzados en Zonas Áridas (CEAZA), La Serena, Chile
8. Department of Physical Geography, Pontificia Universidad Católica de Chile, Santiago, Chile
9. Center for Climate and Resilience Research, Universidad de Chile, Santiago, Chile
10. Universidad Nacional Andrés Bello, Viña del Mar, Chile
11. School of Earth, Energy and Environmental Sciences, Stanford University, Stanford, USA
12. Center for Environmental Remote Sensing, Chiba University, Chiba, Japan
13. Centro Mario Molina, Antonio Bellet 292, Santiago, Chile
14. Harvard School of Public Health (HSPH), Boston, Massachusetts, USA

Table S1. Correlation matrix (Pearson coefficients).

	Mg	Al	Si	P	S	K	Ca	Ti	V	Cr	Mn	Fe	Co	Ni	Cu	Zn	Rb	Sr	Zr	Mo	Ba	Pb
**Mg**	1.00																					
**Al**	0.75	1.00																				
**Si**	0.80	0.97	1.00																			
**P**	-0.15	-0.04	-0.10	1.00																		
**S**	0.45	0.82	0.72	-0.05	1.00																	
**K**	0.73	0.94	0.87	-0.07	0.86	1.00																
**Ca**	0.58	0.55	0.60	0.34	0.28	0.45	1.00															
**Ti**	0.76	0.91	0.87	-0.06	0.81	0.95	0.54	1.00														
**V**	0.67	0.81	0.73	-0.03	0.77	0.93	0.48	0.96	1.00													
**Cr**	0.35	0.61	0.61	-0.12	0.62	0.64	0.31	0.74	0.74	1.00												
**Mn**	0.72	0.77	0.71	0.01	0.69	0.87	0.54	0.94	0.97	0.66	1.00											
**Fe**	0.66	0.77	0.69	-0.03	0.74	0.90	0.44	0.95	0.99	0.73	0.97	1.00										
**Co**	0.61	0.72	0.64	0.02	0.70	0.86	0.46	0.92	0.98	0.73	0.97	0.99	1.00									
**Ni**	0.54	0.84	0.81	-0.03	0.75	0.86	0.36	0.87	0.83	0.85	0.73	0.81	0.78	1.00								
**Cu**	0.56	0.74	0.64	-0.04	0.77	0.90	0.38	0.90	0.98	0.71	0.92	0.97	0.96	0.82	1.00							
**Zn**	0.65	0.75	0.69	0.04	0.75	0.87	0.55	0.91	0.96	0.74	0.94	0.95	0.95	0.80	0.95	1.00						
**Rb**	0.70	0.80	0.73	-0.06	0.78	0.92	0.47	0.95	0.99	0.75	0.96	0.99	0.98	0.83	0.97	0.97	1.00					
**Sr**	0.72	0.91	0.90	-0.09	0.78	0.89	0.65	0.93	0.88	0.77	0.84	0.84	0.82	0.86	0.84	0.90	0.89	1.00				
**Zr**	0.63	0.73	0.69	0.00	0.68	0.82	0.52	0.92	0.95	0.82	0.94	0.96	0.97	0.83	0.92	0.95	0.96	0.87	1.00			
**Mo**	0.54	0.78	0.67	-0.08	0.84	0.93	0.35	0.90	0.96	0.73	0.90	0.95	0.94	0.83	0.98	0.94	0.96	0.85	0.89	1.00		
**Ba**	0.53	0.69	0.61	-0.04	0.70	0.82	0.45	0.90	0.96	0.83	0.92	0.96	0.97	0.82	0.95	0.93	0.95	0.83	0.96	0.93	1.00	
**Pb**	0.61	0.80	0.71	-0.07	0.77	0.91	0.46	0.92	0.97	0.71	0.93	0.94	0.94	0.81	0.97	0.96	0.95	0.90	0.91	0.96	0.92	1.00

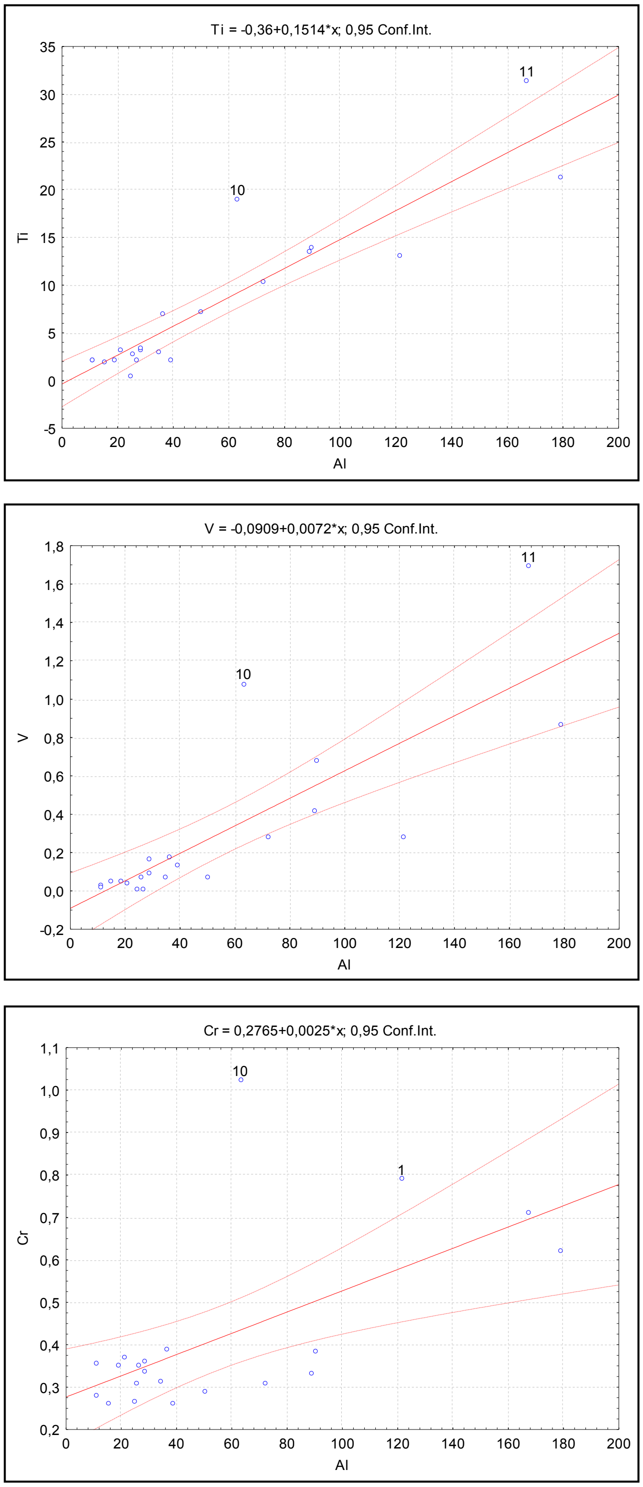

Figure S1. Correlation between the Al concentration and the concentration of Ti, V and Cr. Dotted lines represent the 95% confidence interval. Plots generated by using STATISTICA (data analysis software system, version 7, http://www.statsoft.com/Products/STATISTICA-Features)[38].

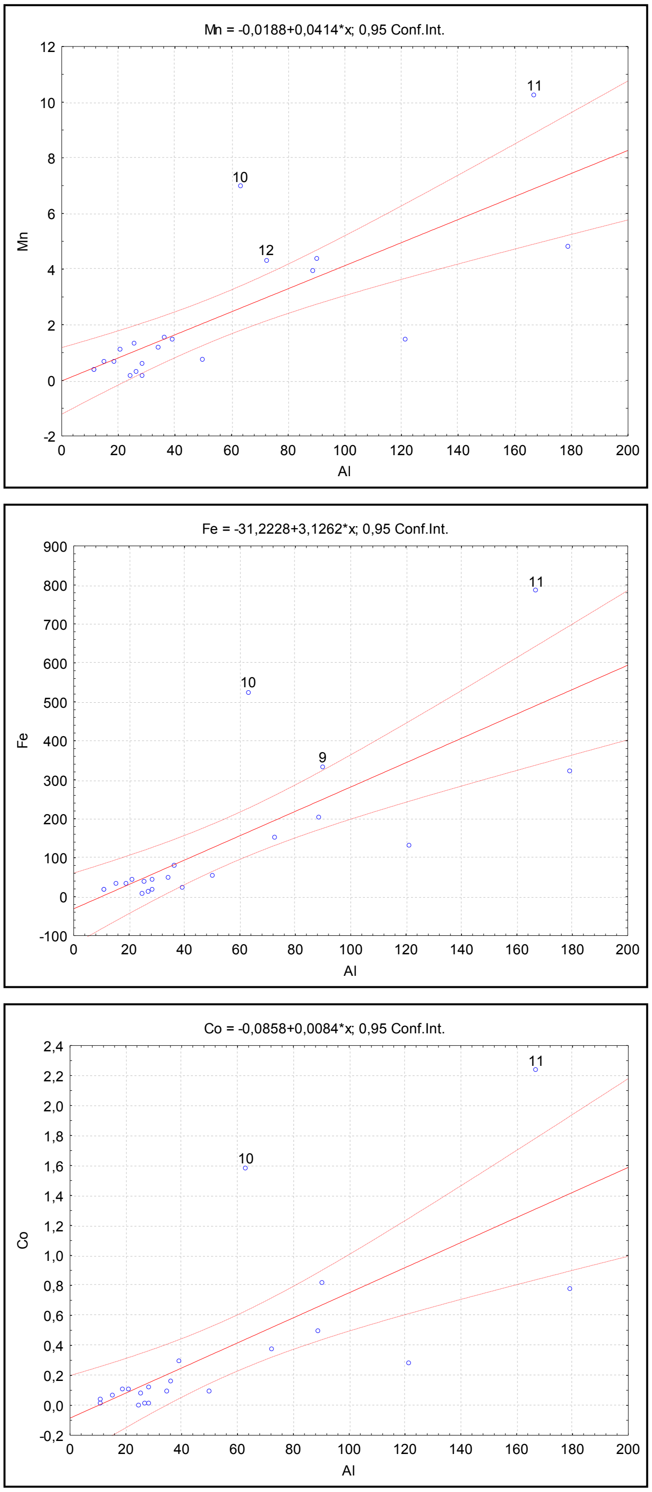

Figure S2. Correlation between the Al concentration and the concentration of Mn, Fe and Co. Dotted lines represent the 95% confidence interval. Plots generated by using STATISTICA (data analysis software system, version 7, http://www.statsoft.com/Products/STATISTICA-Features)[38].

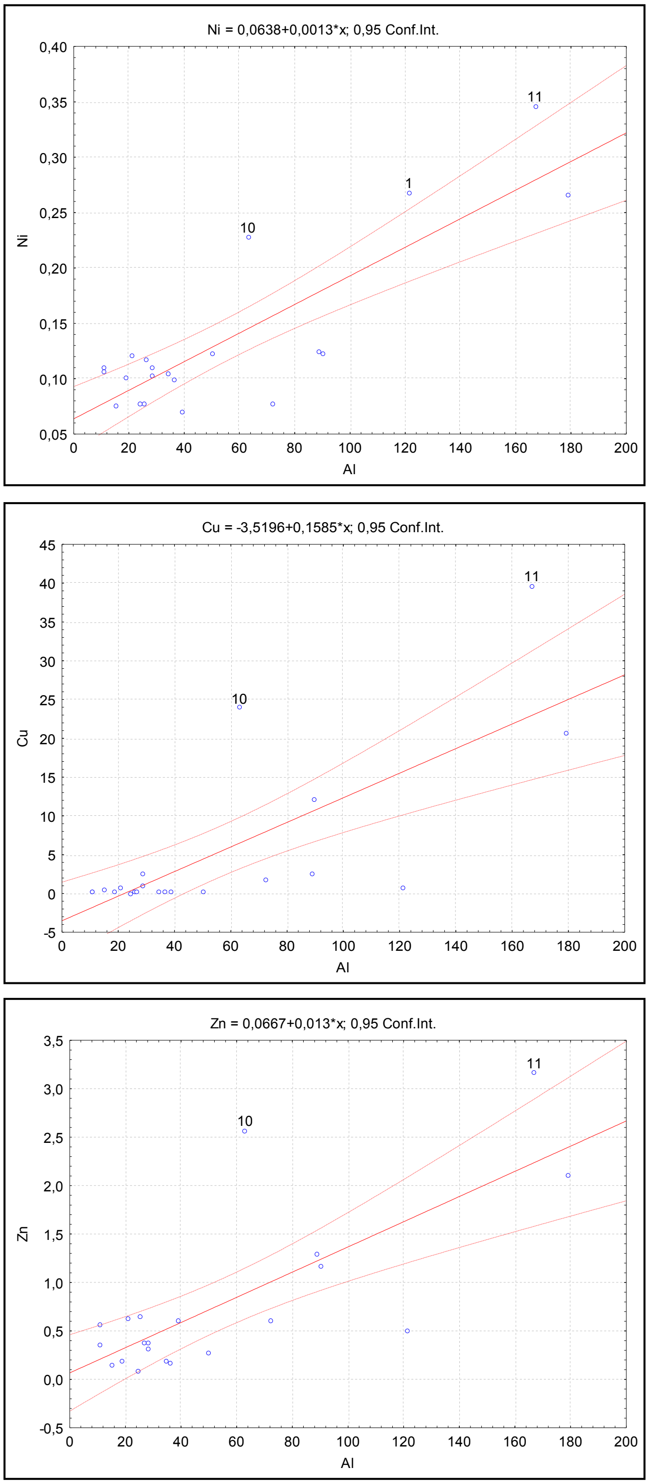

Figure S3. Correlation between the Al concentration and the concentration of Ni, Cu and Zn. Dotted lines represent the 95% confidence interval. Plots generated by using STATISTICA (data analysis software system, version 7, http://www.statsoft.com/Products/STATISTICA-Features)[38].

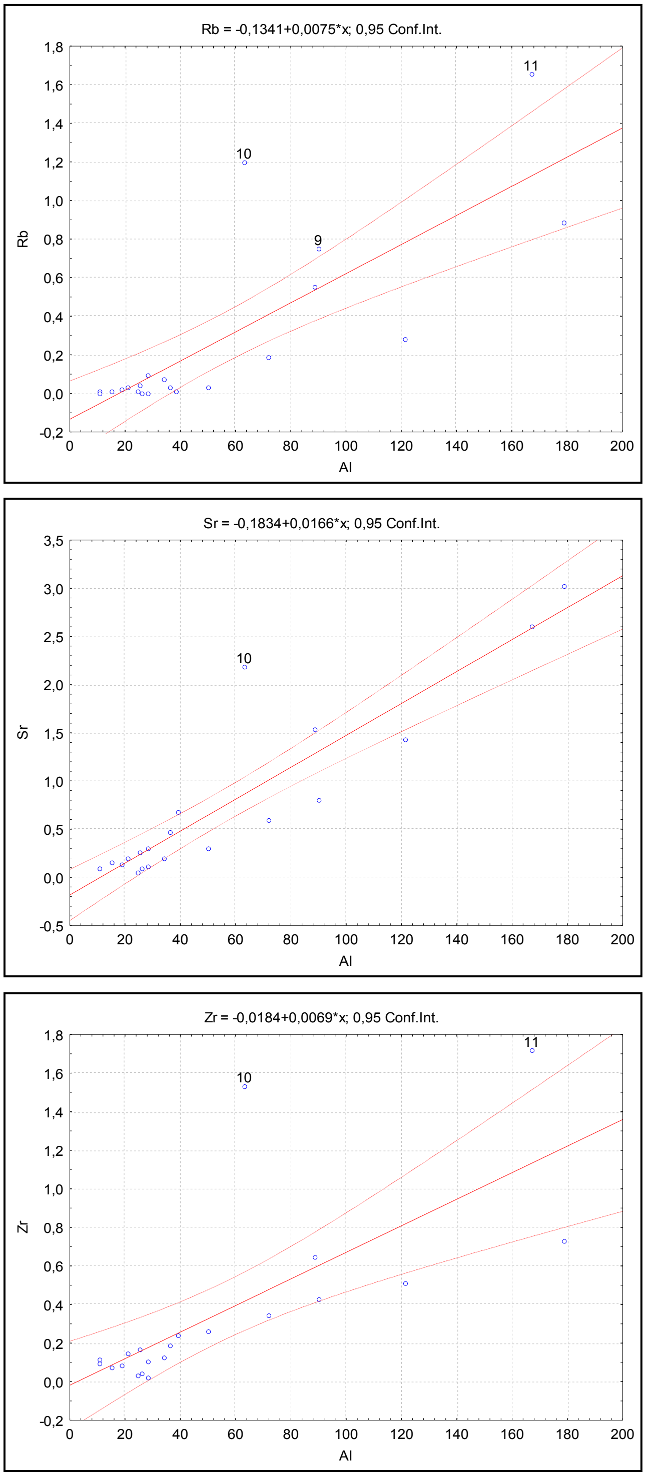

Figure S4. Correlation between the Al concentration and the concentration of Rb, Sr and Zr. Dotted lines represent the 95% confidence interval. Plots generated by using STATISTICA (data analysis software system, version 7, http://www.statsoft.com/Products/STATISTICA-Features)[38].

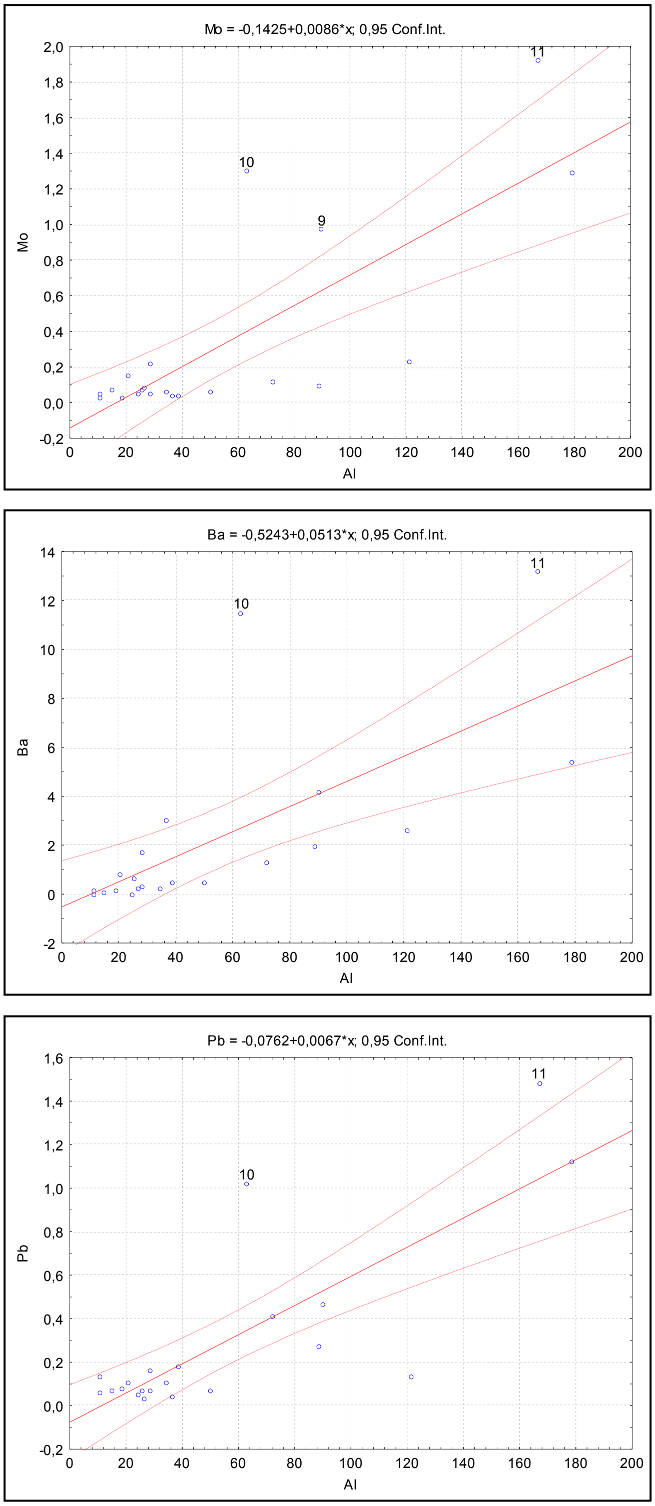

Figure S5. Correlation between the Al concentration and the concentration of Mo, Ba and Pb. Dotted lines represent the 95% confidence interval. Plots generated by using STATISTICA (data analysis software system, version 7, http://www.statsoft.com/Products/STATISTICA-Features)[38].
